# After SARS-CoV-2 Pandemics: New Insights into ICU-Acquired Pneumonia

**DOI:** 10.3390/jcm12062160

**Published:** 2023-03-10

**Authors:** Jean-Francois Timsit

**Affiliations:** 1 Medical and Infectious Diseases ICU, APHP Bichat Hospital F, 75018 Paris, France; jean-francois.timsit@aphp.fr; 2 UMR 1137, IAME, Université Paris Cité, 75018 Paris, France

## 1. Introduction

SARS-CoV-2 pandemics profoundly modified the process of hospital care [1,2]. We will discuss some important findings that have been published in the journal.

## 2. Impact of the Pandemic on the Process of Hospital Care

A continuous demand for assistance and an overcrowded emergency department (ED) requires an optimization of the triage process. Early and safe discharge of low-risk severe acute respiratory syndrome coronavirus 2 (SARS-CoV-2)-infected patients is one of the important elements to analyze. A previously defined physiologic score, called aPNea, composed of the alveolar to arterial oxygen (A–A O_2_) gradient, silent hypoxia diagnosed after a positive 6 min walk test, the importance of lung imaging abnormalities, and the importance of proinflammatory reactions (fever, elevated plasma D-dimer level) compared favorably with traditional severity scores such as NEWS2 (AUC-ROC 0.86 (95% confidence interval (CI), 0.78–0.93)) in predicting a safe hospital discharge from the ED [3]. Further multicenter validation of the aPNea score in Italy and other countries is needed. SARS-CoV-2 pandemics also influenced the prognosis of other patients. In a monocenter cohort study, 11% of the patients who underwent surgery after a femoral fracture were diagnosed as COVID-19 positive after surgery. Mortality, hospital length of stay, and functional outcome were significantly altered by COVID-19 infection. SARS-CoV-2 infection was diagnosed more frequently during the period when less than 80% of health workers were vaccinated [4]. Of course, nosocomial acquisition of SARS-CoV-2 is multifactorial and includes varied contagiousness, prevalence of cases, isolation precautions, use of single rooms, and vaccination of the overall population including family members [5]. However, this finding argues to generalize HCW vaccination and added booster dose.

## 3. Impact of SARS-CoV-2 Pandemics on the Risk of Nosocomial Infection in ICU

SARS-CoV-2 pandemics severely impacted the healthcare system and were responsible for massive breaches in infection control policies.

The risk of nosocomial infection was measured in a multicenter registry of 840 SARS-CoV-2 patients from German hospitals and showed an overall risk of ICU-acquired infection of 40.4%. The risk of bacterial and fungal infections varied widely according to the intensity of oxygenation/ventilation. In patients without mechanical ventilation, bacterial and fungal infections were diagnosed in 17.7% and 0.9% of cases, respectively. The percentage of bacterial/fungal infections reached 23%/6.9% in patients receiving non-invasive ventilation and up to 60.5%/27.5% in ECMO patients [6]. In this study, antimicrobials were used in 88% of cases, more than twice the number of patients actually infected. The overuse of antimicrobials certainly contributed to the high rate of multidrug-resistant infections.

The spread of multidrug-resistant organisms was particularly problematic for Carbapenem-resistant *Acinetobacter baumannii* (CR-Ab). In a multicenter study involving 19 ICUs in Italy, CR-Ab cross colonization occurred in 19% of the patients. CR-Ab colonization was a strong independent predictor of ICU death (OR = 5.463 IC95% 1.572–18.988, *p* = 0.008). Of the 176 Ab carriers, 129 (73%) developed invasive infections, which were mainly VAP and BSI [7].

In fact, VAP is the most important nosocomial infection we have faced during the pandemics. In a retrospective analysis of mechanically ventilated SARS-CoV-2 ARF in France, Moreno et al. reported an incidence of more than 50% [8], which is consistent with the findings of the French multicenter OUTCOME-REA group and a systematic literature review [9]. All studies reported a high risk of VAP, mainly occurring late during mechanical ventilation, due to Staphylococcus aureus, non-fermentative Gram-negative bacteria, and multidrug-resistant Enterobacterales. VAP occurred in the most severe patients, who were more likely to receive corticosteroids, and is associated with low viral clearance [8,9,10].

## 4. Conclusions

Taken together, recent articles suggest that SARS-CoV-2 patients are at high risk for nosocomial infections compared to other mechanically ventilated patients with or without ARDS. Poor infection control practices, local and systemic immune alterations, extremely long duration of mechanical ventilation, and an extensive use of corticosteroids and immunosuppressive agents probably explain this finding, which has a profound impact on prognosis [11]. This finding should be taken into account when prioritizing strategies of preparation for future pandemics.
